# Limited Proteolysis-Coupled Mass Spectrometry Identifies Phosphatidylinositol 4,5-Bisphosphate Effectors in Human Nuclear Proteome

**DOI:** 10.3390/cells10010068

**Published:** 2021-01-04

**Authors:** Martin Sztacho, Barbora Šalovská, Jakub Červenka, Can Balaban, Peter Hoboth, Pavel Hozák

**Affiliations:** 1 Department of Biology of the Cell Nucleus, Institute of Molecular Genetics of the Czech Academy of Sciences, Vídeňská 1083, 142 20 Prague, Czech Republic; martin.sztacho@img.cas.cz (M.S.); can.balaban@img.cas.cz (C.B.); peter.hoboth@img.cas.cz (P.H.); 2 Laboratory of Genome Integrity, Institute of Molecular Genetics of the Czech Academy of Sciences, Vídeňská 1083, 142 20 Prague, Czech Republic; barbora.salovska@img.cas.cz; 3 Laboratory of Applied Proteome Analyses, Institute of Animal Physiology and Genetics of the Czech Academy of Sciences, Rumburská 89, 277 21 Liběchov, Czech Republic; jakub.cervenka@natur.cuni.cz; 4 Microscopy Centre, Institute of Molecular Genetics of the Czech Academy of Sciences, Vídeňská 1083, 142 20 Prague, Czech Republic; 5 Division BIOCEV, Laboratory of Epigenetics of the Cell Nucleus, Institute of Molecular Genetics of the Czech Academy of Sciences, Průmyslová 595, 252 50 Vestec, Czech Republic

**Keywords:** nucleus, limited proteolysis, mass spectrometry, phosphoinositides, phosphatidylinositol 4,5-bisphosphate

## Abstract

Specific nuclear sub-compartments that are regions of fundamental processes such as gene expression or DNA repair, contain phosphoinositides (PIPs). PIPs thus potentially represent signals for the localization of specific proteins into different nuclear functional domains. We performed limited proteolysis followed by label-free quantitative mass spectrometry and identified nuclear protein effectors of the most abundant PIP—phosphatidylinositol 4,5-bisphosphate (PIP2). We identified 515 proteins with PIP2-binding capacity of which 191 ‘exposed’ proteins represent a direct PIP2 interactors and 324 ‘hidden’ proteins, where PIP2 binding was increased upon trypsin treatment. Gene ontology analysis revealed that ‘exposed’ proteins are involved in the gene expression as regulators of Pol II, mRNA splicing, and cell cycle. They localize mainly to non-membrane bound organelles—nuclear speckles and nucleolus and are connected to the actin nucleoskeleton. ‘Hidden’ proteins are linked to the gene expression, RNA splicing and transport, cell cycle regulation, and response to heat or viral infection. These proteins localize to the nuclear envelope, nuclear pore complex, or chromatin. Bioinformatic analysis of peptides bound in both groups revealed that PIP2-binding motifs are in general hydrophilic. Our data provide an insight into the molecular mechanism of nuclear PIP2 protein interaction and advance the methodology applicable for further studies of PIPs or other protein ligands.

## 1. Introduction

The maintenance of genome stability, genome expression, and its inheritance are amongst the main functions of the eukaryotic nucleus. In order to fulfil these functions, the nucleus shows unique, functionally compartmentalized architecture [1]. Nuclear phosphatidylinositol phosphates (PIPs) contribute to the spatial compartmentalization of the nucleus [2,3,4]. PIPs are known regulators of various processes such as membrane architecture and cytoskeletal dynamics, vesicular trafficking, signal transduction, or bone resorption [5,6]. The most abundant phosphatidylinositol 4,5-bisphosphate (PIP2) localizes in the distinct nuclear sub-compartments—nuclear speckles, nucleoli, and nucleoplasmic foci—nuclear lipid islets [2]. In a study of Lewis and co-workers, more than 300 PIP2-interacting proteins involved in chromatin remodeling or RNA processing were identified [7]. PIP2 is also involved in both RNA polymerase I and RNA polymerase II mediated transcription [2,8]. Several nuclear proteins which are linked to the regulation of RNA transcription and mRNA processing have direct PIP2-binding capacity [4,9]. Various limited proteolysis followed by quantitative mass spectrometry (LiP-qMS) approaches have been successfully used for the determination of tertiary structure or conformational changes of proteins [10,11]. The LiP experimental approach uses the effect of ligand in the sample containing the ligand binding proteins. The addition of the ligand into the reaction causes binding of interactors and masks protein binding sites or induces conformational change that affects the accessibility of protease cleavage sites. Nuclear proteins rarely contain the canonical PIPs-binding domains, such as PH, FYVE, or PX domains. Instead, polybasic stretches containing lysine and arginine amino acid residues (K/R motifs) mediate the PIP2 binding [7]. Here we used LiP-qMS to identify the nuclear proteins, which directly bind to PIP2. We took the advantage of the trypsin protease activity, which specifically cleaves the peptide bond at the C-terminus of lysine and arginine residues, and developed LiP-based experimental pipeline using covalently conjugated PIP2 agarose beads followed by qMS to identify the proteins that interact with PIP2. We refer to this experimental pipeline as Phosphoinositide phosphates-based Limited Proteolysis quantitative mass spectrometry (PIPsLiP-qMS). This novel two-step approach allowed us to identify PIP2-interactome of the nuclear proteome and provides data discriminating two different types of PIP2-binding regions. Our approach provides information about structural features of the PIP2–protein interaction on a nuclear proteome scale and a methodological pipeline for further evaluation of other nuclear PIPs interactors and thus determination of their possible functions.

## 2. Materials and Methods

### 2.1. Cell Culture, Antibodies, and Lipid-Conjugated Beads

HeLa cells were cultured in suspension in DMEM media supplemented with 10% fetal bovine serum in spinner flasks at 37 °C 10% CO_2_ atmosphere for 5 days. Antibodies used in this study: anti-GAPDH (Abcam, Cambridge, UK, ab8245), anti-lamin A (Abcam, Cambridge, UK, ab8990), anti-CWC25 (Sigma-Aldrich, St. Louis, MO, USA, HPA062997), anti-MPRIP antibody (Sigma-Aldrich, St. Louis, MO, USA, HPA022901). IRDye^®^ 800 CW Donkey anti-Rabbit IgG (LI-COR Biosciences, Lincoln, NE, USA, 926-32213), IRDye^®^ 800 CW Donkey anti-Mouse IgG (LI-COR Biosciences, Lincoln, NE, USA, 926-32212), PIP2-conjugated agarose beads (Echelon Biosciences Inc., Salt Lake City, UT, USA, P-B045A-2), blocked control agarose beads (Echelon Biosciences Inc., Salt Lake City, UT, USA, P-B000).

### 2.2. Nuclear Lysate Preparation

One liter of HeLa suspension culture was spun at 1300 g at 4 °C for 15 min. Pellet was resuspended in 7 mL of B buffer (50 mM Hepes pH 7.4, 150 mM NaCl, 1 mM DTT) and subjected to Dounce homogenization (20 strokes). Cell nuclei were sedimented by 1800× *g* centrifugation at 4 °C for 5 min. Supernatant was collected as cytoplasmic fraction. Nuclear pellet was washed 4 times in 10 mL of B buffer. Clean nuclear pellet was sonicated in Soniprep 150 (MSE) bench top sonicator (1 s on, 1 s off for 30 cycles at power 10 amplitude microns). Sonicated lysate was spun down at 13,000 g at 4 °C for 15 min. Supernatant was collected as nuclear lysate. Protein concentration was determined by Pierce™ BCA Protein Assay (Thermo Fisher Scientific, Waltham, MA, USA, 23227) according to the manufacturer’s protocol.

### 2.3. Trypsin Inhibition Assay

Five micrograms of bovine serum albumin (BSA; New England Biolabs, Ipswich, MA, USA, B9001S) were diluted in 19 µL of B buffer (50 mM Hepes pH 7.4, 150 mM NaCl, 1 mM DTT). One microliter of trypsin (0.5 µg) (Promega, Madison, WI, USA, V5111) was added to the reaction and incubated at RT for one minute. After one-minute incubation, one microliter of protease inhibitor (cOmplete™, Roche, Basel, Switzerland, 05056489001) was added or reaction was boiled at 100 °C. Protein samples were immediately vortexed and incubated at 4 °C for 60 min to ensure the stability of the reaction. 

### 2.4. PIPsLiP-qMS Experiment

Three liters of HeLa suspension culture were used for nuclear lysate preparation. One milliliter of nuclear lysate with protein concentration of 2.5 mg/mL was used per condition. Eighty microliters of washed PIP2-conjugated agarose beads or control beads respectively, were added into the respective reaction and incubated at 4 °C for 1 h rotating to allow PIP2–protein interaction. After this step, 0.5 µg of trypsin (Promega, Madison, WI, USA, V5111) was added (1:5000 trypsin:total protein ratio), mixed well, and incubated at RT for 1 min. After the incubation, we added 1.4 µL of broad spectrum protease inhibitors (cOmplete™, Roche, Basel, Switzerland, 05056489001) into the reaction, mixed well, and placed on ice. The control beads sample was centrifuged at 300× *g* at 4 °C for 2 min. Supernatant was transferred into a new tube and 80 µL of fresh PIP2-conjugated beads were added into the reaction and the sample was incubated rotating at 4 °C for 1 h. Finally, beads were spun down and washed twice in 1 mL of B buffer and subjected to sample preparation for qMS measurement. PIPsLiP was performed in three independent replicates.

### 2.5. Protein Digestion

Beads were resuspended in 100 mM triethylammonium bicarbonate (TEAB) containing 2% sodium deoxycholate (SDC). Proteins were eluted and cysteines were reduced in one step by heating with 10 mM final concentration of Tris-(2-carboxyethyl)phosphine (TCEP; 60 °C for 30 min). Beads were removed by centrifugation and proteins in the supernatant were incubated with 10 mM final concentration of methyl methanethiosulfonate (MMTS; 10 min RT) to modify reduced cysteine residues. In-solution digestion was performed with 1 µg of trypsin at 37 °C overnight. After digestion, the samples were centrifuged and supernatants were collected and acidified with TFA to 1% final concentration. SDC was removed by ethylacetate extraction [12]. Peptides were desalted using homemade stage tips packed with C18 disks (Empore) according to Rappsilber et al. [13].

### 2.6. Nano Scale Chromatographic Tandem Mass Spectrometry (nLC MS2) Analysis

Nano reversed phase column (EASY Spray column, 50 cm × 75 µm ID, PepMap C18, 2 µm particles, 100 Å pore size) was used for Liquid chromatography-tandem mass spectrometry (LC-MS/MS) analysis. The mobile phase buffer A was composed of water and 0.1% formic acid. The mobile phase B was composed of acetonitrile and 0.1% formic acid. The samples were loaded onto the trap column (Acclaim PepMap 300, C18, 5 µm, 300 Å Wide Pore, 300 µm × 5 mm, 5 Cartridges) at 15 μL/min for 4 min. The loading buffer was composed of water, 2% acetonitrile, and 0.1% trifluoroacetic acid. Peptides were eluted with the mobile phase B gradient from 4% to 35% B in 60 min. Eluting peptide cations were converted to gas phase ions by electrospray ionization and analyzed on a Thermo Orbitrap Fusion (Q OT qIT, Thermo Fisher Scientific, Waltham, MA, USA). Survey scans of peptide precursors from 350 to 1400 *m*/*z* were performed at 120 K resolution (at 200 *m*/*z*) with a 5 × 10^5^ ion count target. Tandem MS was performed by isolation at 1.5 Th with the quadrupole, HCD fragmentation with normalized collision energy of 30, and rapid scan MS analysis in the ion trap. The MS/MS ion count target was set to 10^4^, and the max injection time was 35 ms. Only those precursors with charge state 2–6 were sampled for MS/MS. The dynamic exclusion duration was set to 45 s with a 10 ppm tolerance around the selected precursor and its isotopes. Monoisotopic precursor selection was turned on. The instrument was run in top speed mode with 2 s cycles [14].

### 2.7. Raw Data Processing

Raw data files acquired by LC-MS/MS were processed with MaxQuant v1.6.11.0 [15]. Peak lists were searched against the human SwissProt database (May 2020) using Andromeda search engine [16]. Minimum peptide length was set to seven amino acids, and two missed cleavages were allowed. Dithiomethylation of cysteine was set as a fixed modification while oxidation of methionine and protein N-terminal acetylation were used as variable modifications. Only peptides and proteins with false discovery rate (FDR) lower than 0.01 were accepted. Protein intensities were normalized using MaxLFQ algorithm [17]. MaxQuant output data were further analyzed using Perseus v1.6.12.0 [18] and visualized in R v4.0.0 [19]. Briefly, protein groups identified at the 0.01 FDR level were further filtered to remove potential contaminants, decoys, and proteins identified based on modified peptides only. The resulting matrix was filtered based on the number of missing values (at least three valid values in at least one of the groups), and after log2 transformation, missing values were imputed from normal distribution (width = 0.3 times standard deviation (SD) and shift = 1.8 times SD of the original distribution).

### 2.8. Statistical Analysis

The samples were categorized in two groups—in the first group, LiP was performed in the presence of the PIP2-beads (to protect ‘exposed’-PIP2-binding motifs; PIP2-LiP) while in the second group, LiP was performed in the presence of blank beads, and the supernatant was incubated with PIP2-beads (to reveal ‘hidden’ PIP2-binding motifs; LiP-PIP2). Student’s *t*-test was performed with an additional fold-change cut-off (*p* < 0.05; fold change > 2) to compare differentially enriched proteins between these groups and to identify the most significant hits. Additional false discovery rate (FDR) correction for multiple hypothesis testing was performed (permutation-based FDR 0.05 or 0.1; Perseus v1.6.12.0) and reported in the supplementary files.

### 2.9. Gene Ontology Enrichment

Gene ontology annotation (gene ontology biological process and gene ontology cellular compartment, GOBP and GOCC, resp.) was performed in Perseus, and statistical significance of GOBP and GOCC terms enrichment in the ‘exposed’ and ‘hidden’ groups was evaluated by Fisher’s exact test using all quantified proteins as a background reference set. The complementary GO search for mRNA processing and RNA splicing factors in ‘exposed’ protein group was done using the https://string-db.org/ interface with interaction score set as “medium confidence (0.4)”. For details please refer to STRING web page [20].

### 2.10. Peptide Hydrophobicity Analysis

All identified peptides were assigned a grand average of hydropathy (GRAVY) value (http://www.gravy-calculator.de/index.php). Peptide distributions corresponding to ‘exposed’ proteins, ‘hidden’ proteins, and the remaining proteins were visualized, and the difference between these groups was evaluated using Kruskal–Wallis test. Pairwise comparisons were performed using pairwise Wilcoxon test with Bonferroni correction.

### 2.11. Data Visualization

All plots were generated using R package ‘ggplot2′. The *x*-axis in the volcano plot shows log2-transformed fold change between PIP2-LiP and LiP-PIP2 groups described above; the *y*-axis shows the negative decadic logarithm of the *t*-test *p*-value. In the bubble plots, the *x*-axis shows the proportion of proteins from a GO category from all proteins in the ‘exposed’ or ‘hidden’ group; *y*-axis shows the negative decadic logarithm of *p*-value estimated using Fisher‘s exact test. The size of the bubble corresponds to the number of proteins. In the boxplots, the bold line indicates the median value; box borders represent the 25th and 75th percentiles, and the whiskers represent the minimum and maximum value within 1.5 times of interquartile range. Outliers out of this range are depicted using solid dots.

### 2.12. Bioinformatic Analyses

Sequence retrieval—Canonical protein sequences were obtained from UniProtKB [21] (release 2020_06). Search for PIPs-binding domains—the PROSITE database [22] (release 2019_11) was used for selection of protein domains previously shown to bind to PIPs resulting in 11 protein domains selected: C2; N-terminal C2 (NT-C2); DOCK-homology region (DHR)-1 and -2; epsin NH2-terminal homology (ENTH); Fab1, YOTB/ZK632.12, Vac1 and EEA1 (FYVE); GRAM-like ubiquitin-binding in EAP45 (GLUE); pleckstrin homology (PH); phosphotyrosine interaction domain (PID); phox homology (PX); and Sprouty (SPR) domain. List of all human proteins containing these domains was retrieved from Swiss-Prot [20] using the PROSITE database and compared to the list of the most significant PIP2-interactors. Search for K/R motifs—ScanProsite tool [22] was employed for searches for K/R rich motifs: [KR]-x(3,7)-K-x-[KR]-[KR], [KR]-x(3,7)-K-x-[KR], and [KR]-x(3,7)-K-x-K with match mode set as greedy with no overlaps.

### 2.13. PIP2-Coated Beads Pull-Down and Western Blot

PIP2-coated or control agarose beads (20 µL slurry) were incubated with 1 mg of nuclear lysate for 1 h at 4 °C. Beads were washed 3 times with buffer (50 mM Hepes pH 7.4, 150 mM NaCl, 1 mM DTT) and subjected to western blot analysis. Proteins were loaded on a 4–20% polyacrylamide gel (Bio-Rad Laboratories, Hercules, CA, USA, 4561093), separated by SDS-PAGE, and transferred on a nitrocellulose membrane (BioTrace^TM^, Menlo Park, CA, USA, 66485). Membranes were blocked with 3% BSA in PBS for 1 h and incubated with primary antibodies and with appropriate secondary antibodies conjugated to IRDye. The signal was detected by Odyssey Infrared Imaging System (LI-COR Biosciences, Lincoln, NE, USA).

## 3. Results

### 3.1. Rationale and Experimental Workflow of PIPsLiP-qMS Experiment

**Figure 1:**
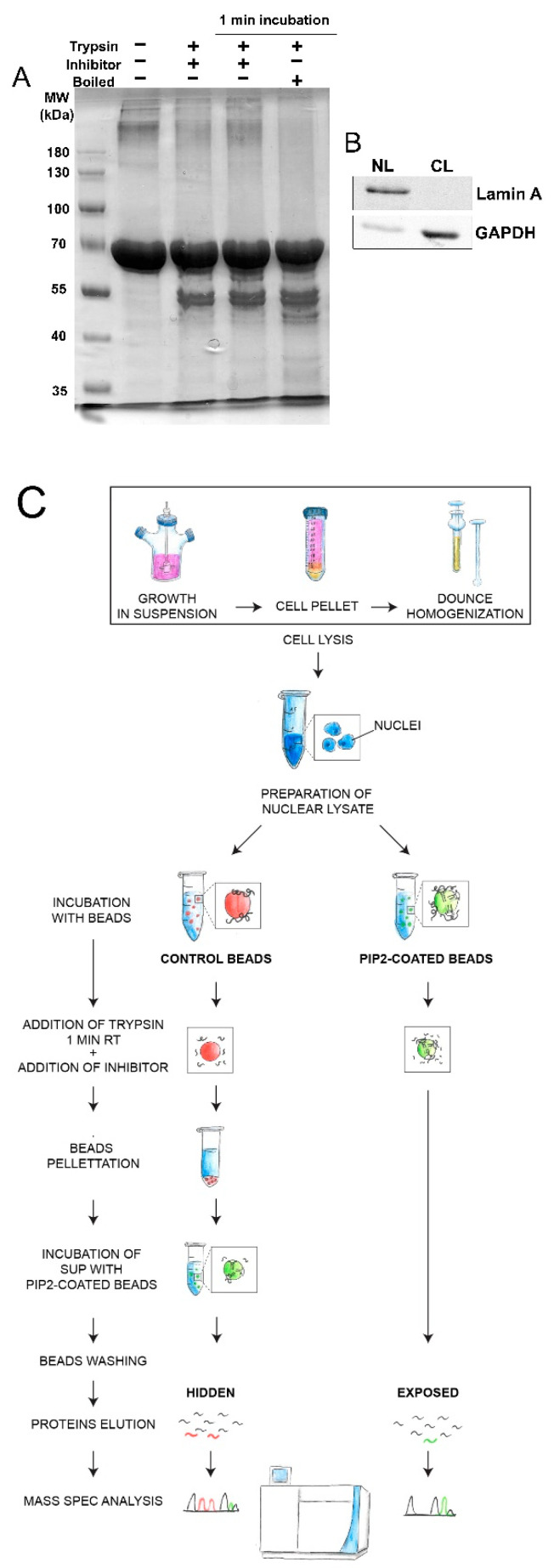
(**A**) Comparison of the trypsin cleavage block efficacy between inhibitor mix and heat inactivation. Bovine serum albumin (BSA) and its cleavage products were Coomassie Brilliant Blue-stained in polyacrylamide gel. First lane (from left) contains only BSA. In second lane, the inhibitors were added to the sample prior to trypsin digestion. Third lane contains a reaction where inhibitors were added after 1 min of trypsin incubation. In fourth lane, the sample was boiled after 1 min of trypsin incubation. All reactions were incubated at 4 °C for additional 1 h to ensure the stability of the trypsin inactivation. (**B**) Western blot (WB) analysis showing the purity of limited proteolysis (LiP) protein lysate starting material. NL—Nuclear lysate, CL—cytoplasmic lysate. WB shows immunodetected nuclear marker lamin A and the cytoplasmic marker GAPDH. (**C**) Scheme of the Phosphoinositide Phosphates based Limited Proteolysis quantitative Mass Spectrometry (PIPsLiP-qMS) experimental workflow. HeLa cells were grown in suspension into high densities followed by centrifugation and Dounce homogenization. The separation of nuclei from cytoplasm was done by centrifugation. Isolated nuclei were sonicated and lysates were prepared. Nuclear lysates were used as starting material in subsequent steps. Phosphatidylinositol 4,5-bisphosphate (PIP2)-coated beads or control beads were added into the nuclear lysate. The control beads served as free surface which might bind proteins non-specifically and thus protect them from trypsin cleavage. In the next step, trypsin was added into both reactions and then it was inhibited after 1 min of incubation at RT. Inactivation was done under non-denaturing conditions (inhibitors), therefore, PIP2-binding capacities of proteins remain persistent. Control beads were collected by centrifugation and discarded from the control sample. The supernatant was transferred into a clean tube and fresh PIP2-covered beads were added and incubated at 4 °C for 1 h. Capacity of proteins to bind PIP2 in this step should be diminished, since the binding sites were not protected by the ligand during LiP. Thus, lower amounts of these proteins will co-precipitate together with PIP2-covered beads. Washed PIP2-beads from both samples were collected; proteins were eluted and subjected to sample preparation for quantitative mass spectrometry analysis.

We developed a novel experimental workflow PIPsLiP-qMS in order to understand molecular processes with involvement of nuclear PIP2. Heat inactivation of trypsin is a necessary step in LiP-qMS. However, this treatment has denaturing effect on all proteins in the sample. Therefore, such approach is not suitable for experiments where additional steps demand proteins in a native conformation. In our experimental set-up, with non-denaturing conditions, we preserve proteins structural folds that are important for recognition and binding of PIP2. The PIP2 pull-down step decreases the complexity of the sample analyzed by qMS. The sample treatment by trypsin is done in the complete nuclear proteome. However, the analysis of only PIP2-bound protein fraction is ensured by pull-down of PIP2-conjugated beads. In order to block trypsin catalyzed cleavage with high efficacy, we used protease inhibitor mix (Roche; see Material Methods), which irreversibly and reversibly inhibits a broad spectrum of proteases. We tested the efficacy of this inhibitor mix to block trypsin cleavage and compared it to the efficacy of heat inactivation standardly used in LiP-MS experiments. We tested the efficacy of trypsin inhibition on BSA cleavage assay (Figure 1A).

BSA cleavage assay revealed that BSA was partially cleaved by trypsin into fragments in all tested conditions. The cleavage occurred at the most accessible sites of BSA. The inhibitors were not able to protect all cleavage sites before reaching full inhibition of trypsin even when inhibitors were added prior to trypsin. Addition of inhibitors to the reaction prior to the addition of trypsin had similar cleavage patterns as the addition of inhibitors after 1 min incubation at RT. More cleavage products at lower molecular weights (approx. 45–60 kDa) and ~25–37 kDa appeared in reaction after heat inactivation, whereas these fragment were almost absent in both reactions with added inhibitors. Based on this, we suggest that the inhibitor mix blocks trypsin cleavage even more efficiently than the heat inactivation. The inhibition (both inhibitors and heat) was effective 1 h after the treatment, which is a necessary pre-condition for subsequent PIP2 pull-down. In the LiP-MS experiment, analysis of complex proteomes remains a challenging task. To overcome this hurdle, in PIPsLiP-qMS we used two steps to decrease the complexity of the analyzed samples. First, for the cell material fractionation, we employed an approach for cell nuclear lysate preparation. It is necessary for the subsequent qMS analysis to have highly concentrated starting material. Our protocol leads to the preparation of highly concentrated nuclear lysates with low cross-contamination. We confirmed the purity of our preparations by Western blot analysis of the nuclear and cytoplasmic markers, lamin A, and GAPDH, respectively (Figure 1B). Second, the covalent ligand conjugation to the agarose beads enabled us to perform the pull-down after trypsin inactivation step (Figure 1C) and thus further decrease the complexity of the samples analyzed by qMS.

### 3.2. The Identification of PIP2-Effectors in Nuclear Proteome by Label-Free qMS

**Figure 2:**
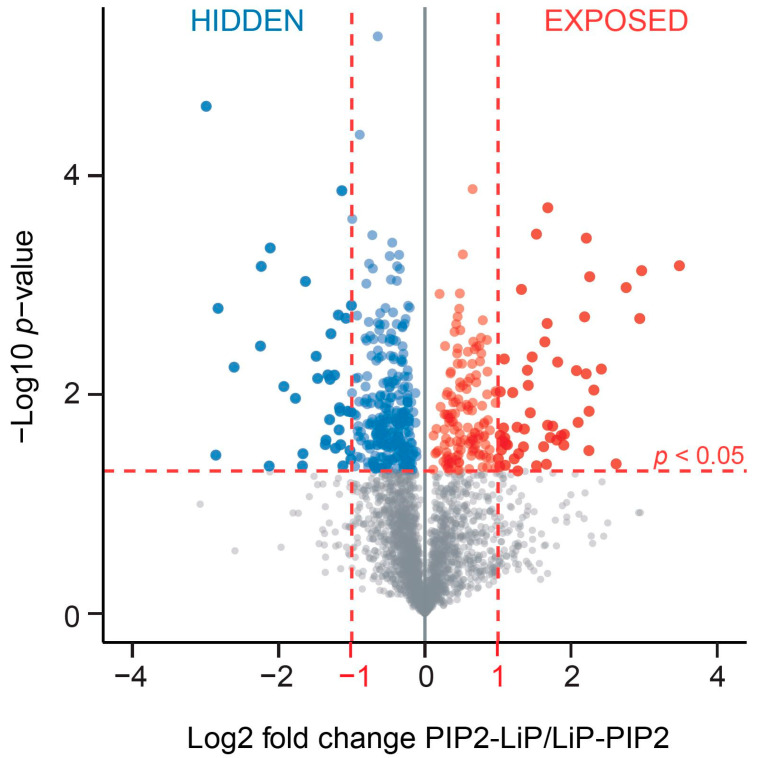
The PIP2-binding proteins differentially identified by PIPsLiP-qMS in HeLa nuclear proteome. LiP was performed in the presence of the PIP2-beads (to protect ‘exposed’ PIP2-binding motifs; PIP2-LiP) or in the presence of blank beads, and then the partially digested supernatant was incubated with PIP2-beads (to reveal ‘hidden’ PIP2-binding motifs; LiP-PIP2). Student’s *t*-test was performed with an additional fold-change cut-off >2 (indicated by vertical red lines) to identify the most significant hits. The blue dots mark proteins enriched in LiP-PIP2 group, while red dots indicate proteins enriched in the PIP2-LiP group.

The qMS-based comparison of the abundance of PIP2-bound proteins between both samples allowed us to identify individual proteins which were associated with PIP2-binding. Our PIPsLiP-qMS experimental approach identified 515 proteins of which PIP2 significantly changed their susceptibility to trypsin cleavage. Such changes might be due to direct PIP2-binding and thus masking cleavage site or indirect due to the conformational change which in turn exposes or hides trypsin recognition site. Therefore, these proteins can be divided into two groups. In the first group, the addition of PIP2 led to the protection of proteins from trypsin cleavage and thus their PIP2-coprecipitation was increased. This group includes direct interactors and also the proteins which do not have any PIP2-binding capacity, but their conformation is indirectly changed through interactions with components of the PIP2-binding protein complexes. We refer to this group of 191 proteins as ‘exposed’. In this set of proteins, PIP2 potentiates the formation of a complex or an indirect conformational change leading to cleavage protection by burying of trypsin cleavage sites. The second protein group, that we refer to as ‘hidden’, contains proteins that showed increased PIP2-binding upon trypsin treatment. The ‘hidden’ group includes 324 proteins in which trypsin cleavage promotes the PIP2-binding sites accessibility. We discriminated between ‘exposed’ and ‘hidden’ PIP2-interactors by further analyzing the significant PIP2-effectors in nuclear proteome and set the minimal change of protein abundance to 2-fold (Figure 2 and Supplementary Table S1).

Taken together, here we identified the nuclear PIP2-interacting proteins and proteins whose PIP2 interactions change their conformation. We further refer to the proteins from both groups as nuclear PIP2-effectors. We further aimed to identify processes in which these proteins participate and their sub-nuclear localization.

### 3.3. PIP2-Effectors in Human Nuclear Proteome Are Mainly Linked to Regulation of Gene Expression

**Figure 3:**
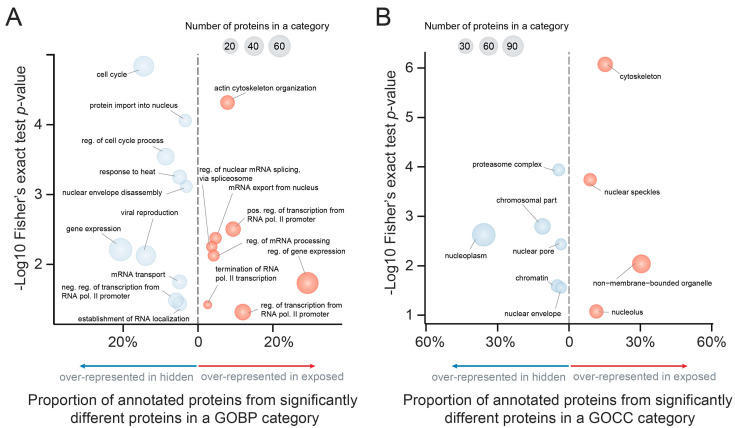
Gene ontology analysis of PIP2-effectors based on (**A**) biological process (GOBP) and (**B**) cellular compartment (GOCC). Bubble charts represent ‘exposed’ and ‘hidden’ proteins using all quantified proteins as a background reference set. *X*-axis shows the proportion of proteins from a gene ontology (GO) category from all proteins in the ‘exposed’ (red) or ‘hidden’ (blue) group. *Y*-axis shows the -log10 *p*-value estimated using Fisher’s exact test. The size of the bubble corresponds to number of proteins.

Gene ontology (GO) over-representation analysis of ‘exposed’ and ‘hidden’ proteins provided insight into the function of PIP2 in the cell nucleus. In this analysis, we identified biological processes where ‘exposed’ and ‘hidden’ PIP2-effector proteins participate (Figure 3A and Supplementary Table S2; GOBP).

Moreover, GO analysis provided us with the information about the localization of the processes into the various nuclear sub-compartments (Figure 3B and Supplementary Table S2; GOCC). PIP2-effector localization overlaps with already known nuclear PIP2 pools—nuclear speckles, nucleoli, non-membrane bound organelles, and also cytoskeleton. The ‘exposed’ protein group is linked with regulation of polymerase II mediated transcription, mRNA processing, and actin organization, whereas ‘hidden’ group of proteins belongs to later stages of gene expression such as mRNA transport, RNA localization, or peripheral processes connected to the nuclear envelope or nuclear protein import. Therefore, ‘exposed‘ and ‘hidden‘ proteins have different sub-nuclear localizations, which suggests that ‘exposed‘ and ‘hidden‘ proteins participate in different nuclear processes.

### 3.4. PIP2-Binding Protects Hydrophilic Regions

**Figure 4:**
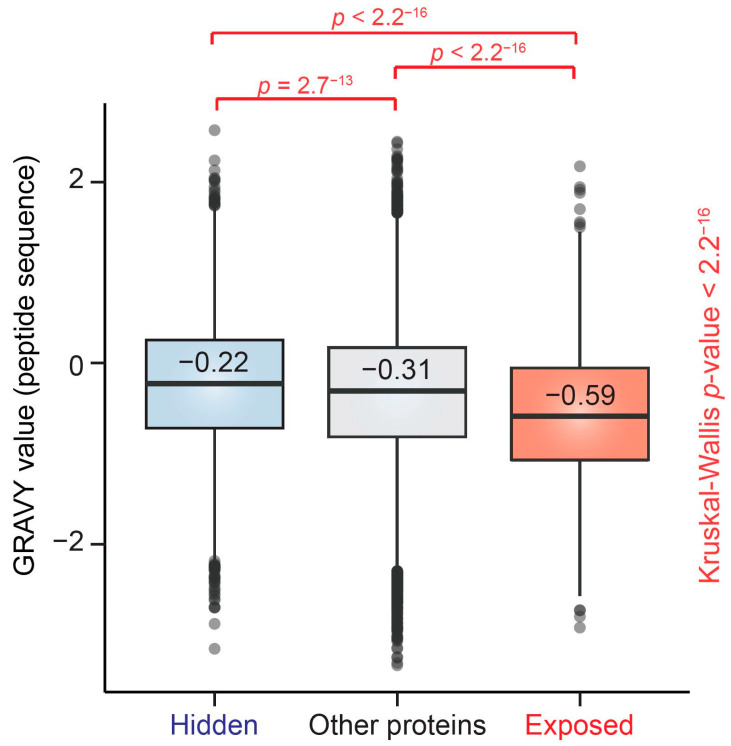
Hydropathy index distribution of peptides corresponding to ‘exposed’ and ‘hidden’ protein groups. GRAVY value distributions of identified peptides corresponding to ‘exposed’ proteins, ‘hidden’ proteins, and the remaining proteins from the data set (‘Other proteins’) are shown. The difference between these groups was evaluated using Kruskal–Wallis test. Pairwise comparisons were performed using pairwise Wilcoxon test with Bonferroni correction. The hydropathy values range from −2 to +2 for most proteins, with the positively rated proteins being more hydrophobic. The bold line and the number above the line indicate median values; box borders represent the 25th and 75th percentiles, and whiskers represent the minimum and maximum value within 1.5 times of interquartile range. Outliers out of this range are depicted using solid dots.

Our GO analysis revealed functional and localization differences between proteins that belong to ‘exposed’ and ‘hidden’ PIP2-effectors. We further asked whether these differences are due to the variance in PIP2-binding region properties. Therefore, we performed a bioinformatic analysis of the hydropathy index of amino acid residues within peptides detected by PIPsLiP (Figure 4).

This analysis revealed the difference in PIP2-binding effect on the protection of protein regions based on their hydropathy. First class of proteins showed higher degree of binding with exposed PIP2-binding regions. The peptides bound to PIP2-covered beads showed the highest hydrophilicity of their side chains. This suggests that these regions are not structural parts of hydrophobic cores of globular protein domains. On the contrary, the peptides belonging to the ‘hidden’ group showed lower hydrophilic properties than both ‘exposed’ and control protein groups. These data are in agreement with our hypothesis that ‘hidden’ regions are not primarily accessible to PIP2 ligand, but become accessible upon the conformational change. In summary, these data suggest that nuclear PIP2-associated effectors belong to at least two functional classes and that PIP2-binding regions are likely situated in the unstructured motifs. Importantly, the interacting motifs of ‘exposed’ proteins are hydrophilic, suggesting that they interact with phosphorylated head groups of PIP2 and not hydrophobic acyl group of this lipid. Therefore, we suggest that the differentially phosphorylated head groups of different PIPs represent an important signal for the functional localization of the interacting nuclear proteins.

### 3.5. PIP2-Effector Proteins Contain Polybasic K/R Motifs

The majority of PIP2-associated nuclear effectors do not contain the canonical PIPs binding domains [7]. The PIPsLiP-qMS approach identified only two proteins with PIPs-binding PH domain amongst the most significant PIP2-interactors (Supplementary Table S3). The low incidence of canonical PIPs-binding domains in both ‘exposed’ and ‘hidden’ proteins is in agreement with previous observations and thus suggests that interactions between PIP2 and nuclear proteins are mediated by other regions [7]. We analyzed the abundances of polybasic K/R-rich motifs responsible for PIP2-binding. We searched for K/R motif K/R-x(3-7)-K-x-K/R-K/R and related K/R-x(3-7)-K-x-K/R and K/R-x(3-7)-K-x-K (see Section 2.12. for details). Motif K/R-x(3-7)-K-x-K/R-K/R is present in 25% of ‘exposed’ and 24% of ‘hidden’ proteins. Motif K/R-x(3-7)-K-x-K/R is in 66% of ‘exposed’ and 57% of ‘hidden’ proteins. Motif K/R-x(3-7)-K-x-K is in 59% of ‘exposed’ and 41% of ‘hidden’ proteins (Supplementary Tables S4 and S5). Based on these results we assume that both, ‘exposed’ and ‘hidden’ proteins, might interact with PIP2 via these K/R motifs. Proteins without these motifs possibly have another unknown PIP2-binding motif or may interact with PIP2-binding protein complexes.

## 4. Discussion

PIPs are important functional regulators of mammalian nuclear processes [4]. PIPs are protein ligands with the ability to recruit and thus affect the localization, conformation, and activity of their protein effectors [4,9,23]. Therefore, it is of particular interest to identify the PIP2 nuclear effectors. Based on the PIP2-associated proteome, we can predict the molecular processes in which PIP2-effectors participate. Affinity purification in combination with MS analysis is, due to its simplicity, the method of choice for the identification of the effectors of any molecule. However, this method is often obstructed by constrains of high sample complexity, non-specific binding of proteins to bait or dynamic range of the sample, leading to relatively high degree of false positives [24]. The balance between the reduction of the protein complexity and enrichment of the target pool of proteins are the essentials for the protein extract preparation [7]. Our experimental PIPsLiP-qMS pipeline obviates the problems with dynamic range and specimen complexity, since trypsin cleavage in combination with cell fractionation and PIP2 pull-down reduce the antagonizing effect of the most abundant proteins within the sample for subsequent qMS analysis [24]. Integration of LiP with qMS provides a powerful tool to determine the boundaries of structured protein domain with an unprecedented accuracy [10]. However, the standard LiP-MS approach demands heat inactivation of the protease and therefore is not suitable for our subsequent affinity purification step, where proteins need to be in the native non-denatured state [25]. In our approach, we eliminated this obstacle by introducing protease inhibitors, which is important for the identification of the proteins in their native conformation.

In the study of Lewis and co-workers, the authors used the neomycin extraction which might not provide the entire range of PIPs-effector proteins [7]. Therefore, we developed an alternative experimental approach to expand these data and correlate both methods. The cross-correlation between results obtained by both approaches suggests that each approach leads to a different set of analyzed proteins (Supplementary Figure S1). However, the comparison of results from GO analysis provides a similar set of participating nuclear processes such as gene expression, RNA processing, and chromatin regulation [7]. This is in agreement with our previous findings that PIP2 regulates both Pol I and Pol II mediated transcription, epigenetic regulation and RNA processing [2,8,9,26]. We validated PIPsLiP-qMS approach by control pull-down experiments followed by WB detection of two chosen proteins MPRIP and CWC25 and showed that indeed both proteins have the binding capacity for PIP2 (Supplementary Figure S2). Furthermore, ~75% of identified proteins from the most significant hits are annotated as nuclear [20] or show nuclear signal detected by immunofluorescence at www.proteinatlas.org database [27,28] (Supplementary Table S6).

GO analysis of PIP2-effectors identified proteins linked to the nuclear speckles. Nuclear speckles are enriched in mRNA splicing factors and supply them to the active splicing sites [29,30]. Importantly, nuclear speckles are sites of highest PIP2 concentration in the cell nucleus [2]. Our present data support the notion of a regulatory axis between PIP2 and mRNA splicing regulation [31]. Furthermore, our PIPsLiP-qMS approach identified proteins associated with mRNA processing, mRNA splicing, and some proteins linked to PI-PLCβ1 interactome [31] (Supplementary Table S7 and Supplementary Figure S3). PI-PLCβ1 is a nuclear enzyme that catalyzes the cleavage of PIP2 and is a key factor which regulates splicing factor SRSF3 and skeletal muscle differentiation [31,32]. Therefore, we suggest that the PIP2 level at nuclear speckles regulates localization of splicing factors that bind PIP2 and thus their availability for the active splicing events [33]. In this model, PIP2 levels at nuclear speckles would represent an important determinant of the mRNA splicing rate in eukaryotic cell. 

GO of PIP2-enriched ‘exposed’ proteins shows significant increase of the regulators of Pol II transcription, gene expression, and RNA processing, whereas ‘hidden’ proteins belong to the nuclear pore complex and RNA export and therefore could regulate later stages of the gene expression. In agreement with this, our bioinformatic search from the GOCC database revealed proteins based on particular sub-cellular localization and indicated that ‘hidden’ proteins localize to the nuclear periphery. PIPsLiP-qMS provided data which link ‘exposed’ PIP2-effectors and actin cytoskeleton. The growing body of evidence supports the key role of actin and cytoskeleton-related proteins in the establishment of nuclear architecture competent for gene expression [34,35]. Our laboratory identified first nuclear actin motor protein nuclear myosin 1 (NM1), which functions as Pol II transcription factor [36,37,38]. The importance of actin in the activity of Pol I and Pol II has also been documented [37,39]. Therefore, the presented PIPsLiP-qMS identification of the PIP2-effectors associated with actin cytoskeleton is in agreement with the previous studies that showed involvement of nucleoskeleton in the regulation of gene expression [37,38,39,40,41]. Moreover, the novel PIP2-binding actin- and cytoskeleton-related proteins identified here are interesting candidates for further studies.

GO analysis of PIP2-effectors further identified proteins linked to proteasome, heat response, and nucleoli localization. Heat shock induced protein aggregates or misfolded proteins are directed to nucleoli [42,43]. The degradation of protein aggregates is in general mediated by proteasomal or autophagy processes [44,45,46]. Ubiquitinated and heat shock proteins form aggresomes within the nucleolus [43,47] Proteins sensitive to heat shock denaturation, which are enriched for disordered regions, can be refolded within the nucleolus [42]. Nucleolar aggresomes contain proteins such as fibrillarin, ubiquitin, or autophagy factor LC3 [48,49]. In yeast, a starvation-mediated autophagy specifically removes some nucleolar factors [50]. Our PIPsLiP approach identified key regulator of autophagy serine-threonine kinase mammalian target of rapamycin (mTOR) as a PIP2-effector protein. mTOR localizes to nucleolus and is implicated in nucleolar processes such as RNA polymerase I and III -mediated transcription [51,52]. Moreover, the nucleolar protein nucleophosmin is involved in non-canonical autophagy when Pol I is inhibited [53]. PIP2 is a nucleolar factor affecting the activity of nucleolar proteins such as Pol I and fibrillarin [8,26]. When ubiquitin proteasome system is inhibited, the fibrillarin colocalizes in nucleolar aggregates with ubiquitin and LC3 [49]. The nucleolus thus represents a regulatory hub for protein quality control through ubiquitin proteasome system and autophagy signaling. Therefore, our data that identified nucleolar PIP2-interactors are in agreement with the previous notion of the nucleolar involvement in the regulation of autophagy.

GO analysis also identified viral infection response proteins. Viruses often hijack the transcription regulatory processes in order to replicate. In this regard, the recent work of Prof. Akgül laboratory represents the interesting connection of nuclear PIP2 levels increase upon HPV infection [54]. Our GO data presented here are in accordance with Akgül and coworkers, suggesting that PIP2-dependent nuclear pathway might represent promising candidate for regulation of viral infection surveillance. Therefore, the involvement of ‘hidden’ proteins in the later stages of gene expression and acting downstream of the ‘exposed’ group points to their effector role in the mRNA export, response to heat or viral replication. Furthermore, based on their localization, it seems that they act in different sites of the nucleus than ‘exposed’ group of proteins, indicating the mobility of PIP2-associated effectors from the sites of their primary action to other sub-nuclear compartments.

PIPsLiP-qMS data analysis further describes the hydropathy properties of regions responsible for the PIP2 interaction. Our hydropathy data show that PIP2-binding motifs are presumably situated in the regions with increased hydrophilicity. Our subsequent bioinformatic search found polybasic K/R motifs in a substantial portion of PIPsLiP-qMS identified proteins.

In summary, we developed the PIPsLiP-qMS experimental pipeline which enabled the identification of nuclear PIP2-effectors. Data provided here determine the nuclear PIP2-effectors which uniquely belong to two protein groups representing regulators of such processes as gene expression, Pol II transcription, and mRNA splicing. These protein groups show different sub-nuclear locations of their actions. Further, we discovered that the PIP2-binding motifs are hydrophilic and thus presumably interact with inositol head group. The PIPsLiP-qMS thus represents a promising approach for identification of ligand–effector interactions and further determination of specific binding motifs. Standard affinity-based MS approaches do not allow for the characterization of protein topography at the site of ligand-protein association. In contrast, the proteolytic cleavage which preferentially occurs at accessible, unstructured, and flexible regions of native proteins, allows for the discrimination and definition of these regions, since globular domains are typically resistant to proteolysis [11]. Therefore, PIPsLiP-qMS provides novel information about the topology, organization, and conformational changes of proteins associated with PIP2 binding. Moreover, the combination of other proteases may provide a better dissection of ligand binding sequences in future studies, e.g., non-specific proteinase K which would enable a more general view of the exposed regions. This experimental pipeline is theoretically applicable for any other ligand which is covalently linked to beads. Our high-throughput PIPsLiP-qMS approach provides a powerful, label-free tool to study protein–lipid interactions and to elucidate the molecular functions of nuclear PIPs and their effectors. 

## Data Availability

Data is contained within the article or Supplementary Materials.

## Supplementary Materials

The following files are available online at https://www.mdpi.com/2073-4409/10/1/68/s1, Table S1: Summary of all protein groups identified in this study; Table S2: Selected results from the gene ontology overrepresentation analysis; Table S3: The PIP2-binding domain-containing proteins identified by bioinformatic analysis; Table S4: The summary of bioinformatic analysis of PIP2-binding motifs in ‘exposed’ protein group; Table S5: The summary of bioinformatic analysis of PIP2-binding motifs in ‘hidden’ protein group; Table S6: Summary of the most significant hits from the ‘exposed’ and ‘hidden’ proteins including the information about protein nuclear localization; Table S7: The list of proteins annotated in GOBP as mRNA splicing, mRNA processing factors, and protein linked to nuclear speckles; Figure S1: The Venn diagram representing the overlap of data provided by PIPsLiP-qMS with data presented in [7]; Figure S2: The verification of PIP2-binding capacity of MPRIP and CWC25; Figure S3: The Gene ontology analysis of ‘Exposed’ protein group using STRING.

## References

[1] Dundr M., Misteli T. (2001). Functional architecture in the cell nucleus. Biochem. J..

[2] Sobol M., Krausová A., Yildirim S., Kalasová I., Fáberová V., Vrkoslav V., Philimonenko V., Marášek P., Pastorek L., Čapek M. (2018). Nuclear phosphatidylinositol 4,5-bisphosphate islets contribute to efficient RNA polymerase II-dependent transcription. J. Cell. Sci..

[3] Sztacho M., Sobol M., Balaban C., Eliana S., Lopes E., Hozák P. (2019). Nuclear phosphoinositides and phase separation: Important players in nuclear compartmentalization. Adv. Biol. Regul..

[4] Castano E., Yildirim S., Fáberová V., Krausová A., Uličná L., Paprčková D., Sztacho M., Hozáket P. (2019). Nuclear phosphoinositides-versatile regulators of genome functions. Cells.

[5] Di Paolo G., De Camilli P. (2006). Phosphoinositides in cell regulation and membrane dynamics. Nature.

[6] Sztacho M., Segeletz S., Sanchez-Fernandez M.A., Czupalla C., Niehage C., Hoflack B. (2016). BAR proteins PSTPIP1/2 regulate podosome dynamics and the resorption activity of osteoclasts. PLoS ONE.

[7] Lewis A.E., Sommer L., Arntzen M.Ø., Strahm Y., Morrice N.A., Divecha N., D‘Santos C.S. (2011). Identification of nuclear phosphatidylinositol 4,5-bisphosphate-interacting proteins by neomycin extraction. Mol. Cell. Proteom..

[8] Yildirim S., Castano E., Sobol M., Philimonenko V.V., Dzijak R., Venit T., Hozák P. (2013). Involvement of phosphatidylinositol 4,5-bisphosphate in RNA polymerase I transcription. J. Cell. Sci..

[9] Ulicna L., Kalendova A., Kalasova I., Vacik T., Hozák P. (2018). PIP2 epigenetically represses rRNA genes transcription interacting with PHF8. Biochim. Biophys. Acta Mol. Cell. Biol. Lipids.

[10] Suh M.J., Pourshahian S., Limbach P.A. (2007). Developing limited proteolysis and mass spectrometry for the characterization of ribosome topography. J. Am. Soc. Mass. Spectrom..

[11] Gao X., Bain K., Bonanno J.B., Buchanan M., Henderson D., Lorimer D., Marsh C., Reynes J.A., Sauder J.M., Schwinn K. (2005). High–throughput limited proteolysis/mass spectrometry for protein domain elucidation. J. Struct. Funct. Genom..

[12] Masuda T., Tomita M., Ishihama Y. (2008). Phase transfer surfactant-aided trypsin digestion for membrane proteome analysis. J. Proteome Res..

[13] Rappsilber J., Mann M., Ishihama Y. (2007). Protocol for micro-purification, enrichment, pre-fractionation and storage of peptides for proteomics using stagetips. Nat. Protoc..

[14] Hebert A.S., Richards A.L., Bailey D.J., Ulbrich A., Coughlin E.E., Westphall M.S., Coon J.J. (2014). The one hour yeast proteome. Mol. Cell. Proteom..

[15] Cox J., Mann M. (2008). MaxQuant enables high peptide identification rates, individualized p.p.b.-range mass accuracies and proteome-wide protein quantification. Nat. Biotechnol..

[16] Cox J., Neuhauser N., Michalski A., Scheltema R.A., Olsen J.V., Mann M. (2011). Andromeda: A peptide search engine integrated into the MaxQuant environment. J. Proteome Res..

[17] Cox J., Hein M.Y., Luber C.A., Paron I., Nagaraj N., Mann M. (2014). Accurate proteome-wide label-free quantification by delayed normalization and maximal peptide ratio extraction, termed MaxLFQ. Mol. Cell. Proteom..

[18] Tyanova S., Temu T., Sinitcyn P., Carlson A., Hein M.Y., Geiger T., Mann M., Cox J. (2016). The Perseus computational platform for comprehensive analysis of (prote)omics data. Nat. Methods.

[19] R Core Team (2020). R: A Language and Environment for Statistical Computing.

[20] Szklarczyk D., Gable A.L., Lyon D., Junge A., Wyder S., Huerta-Cepas J., Simonovic M., Doncheva N.T., Morris J.H., Bork P. (2019). STRING v11: Protein-protein association networks with increased coverage, supporting functional discovery in genome-wide experimental datasets. Nucleic Acids Res..

[21] UniProt C. (2019). UniProt: A worldwide hub of protein knowledge. Nucleic Acids Res..

[22] Sigrist C.J.A., de Castro E., Cerutti L., Cuche B.A., Hulo N., Bridge A., Bougueleret L., Xenarios I. (2013). New and continuing developments at prosite. Nucleic Acids Res..

[23] Faberova V., Kalasová I., Krausová A., Hozák P. (2020). Super-resolution localisation of nuclear PI(4)P and identification of its interacting proteome. Cells.

[24] Zubarev R.A. (2013). The challenge of the proteome dynamic range and its implications for in-depth proteomics. Proteomics.

[25] Schopper S., Kahraman A., Leuenberger P., Feng Y., Piazza I., Müller O., Boersema P.J., Picotti P. (2017). Measuring protein structural changes on a proteome-wide scale using limited proteolysis-coupled mass spectrometry. Nat. Protoc..

[26] Guillen-Chable F., Corona U.R., Pereira-Santana A., Bayona A., Rodríguez-Zapata L.C., Aquino C., Šebestová L., Vitale N., Hozak P., Castano E. (2020). Fibrillarin ribonuclease activity is dependent on the gar domain and modulated by phospholipids. Cells.

[27] Uhlen M., Fagerberg L., Hallström B.M., Lindskog C., Oksvold P., Mardinoglu A., Sivertsson A., Kampf C., Sjöstedt E., Navani S. (2015). Proteomics. Tissue-based map of the human proteome. Science.

[28] Thul P.J., Akesson L., Wiking M., Mahdessian D., Geladaki A., Blal H.A., Alm T., Asplund A., Björk L., Breckels L.M. (2017). A subcellular map of the human proteome. Science.

[29] Lamond I.A., Spector D.L. (2003). Nuclear speckles: A model for nuclear organelles. Nat. Rev. Mol. Cell. Biol..

[30] Mintz P.J., Patterson S.D., Neuwald A.F., Spahr C.S., Spector D.L. (1999). Purification and biochemical characterization of interchromatin granule clusters. EMBO J..

[31] Bavelloni A., Faenza I., Cioffi G., Piazzi M., Parisi D., Matic I., Maraldi N.M., Cocco L. (2006). Proteomic-based analysis of nuclear signaling: PLCbeta1 affects the expression of the splicing factor SRp20 in Friend erythroleukemia cells. Proteomics.

[32] Faenza I., Ramazzotti G., Bavelloni A., Fiume R., Gaboardi G.C., Follo M.Y., Gilmour R.S., Martelli A.M., Ravid K., Cocco L. (2007). Inositide-dependent phospholipase C signaling mimics insulin in skeletal muscle differentiation by affecting specific regions of the cyclin D3 promoter. Endocrinology.

[33] Hochberg-Laufer H., Neufeld N., Brody Y., Nadav-Eliyahu S., Ben-Yishay R., Shav-Tal Y. (2019). Availability of splicing factors in the nucleoplasm can regulate the release of mRNA from the gene after transcription. PLoS Genet..

[34] Viita T., Kyheröinen S., Prajapati B., Virtanen J., Frilander M.J., Varjosalo M., Vartiainen M.K. (2019). Nuclear actin interactome analysis links actin to KAT14 histone acetyl transferase and mRNA splicing. J. Cell Sci..

[35] Yamazaki S., Yamamoto K., Harata M. (2015). Contribution of nuclear actin to transcription regulation. Genom. Data.

[36] Pestic-Dragovich L., Stojiljkovic L., Philimonenko A.A., Nowak G., Ke Y., Settlage R.E., Shabanowitz J., Hunt D.F., Hozak P., de Lanerolle P. (2000). A myosin I isoform in the nucleus. Science.

[37] Philimonenko V.V., Zhao J., Iben S., Dingová H., Kyselá K., Kahle M., Zentgraf H., Hofmann W.A., de Lanerolle P., Hozák P. (2004). Nuclear actin and myosin I are required for RNA polymerase I transcription. Nat. Cell. Biol..

[38] Hofmann W.A., Vargas G.M., Ramchandran R., Stojiljkovic L., Goodrich J.A., de Lanerolle P. (2006). Nuclear myosin I is necessary for the formation of the first phosphodiester bond during transcription initiation by RNA polymerase II. J. Cell Biochem..

[39] Hofmann W.A., Stojiljkovic L., Fuchsova B., Vargas G.M., Mavrommatis E., Philimonenko V., Kysela K., Goodrich J.A., Lessard J.L., Hope T.J. (2004). Actin is part of pre-initiation complexes and is necessary for transcription by RNA polymerase II. Nat. Cell Biol..

[40] Takahashi Y., Hiratsuka S., Machida N., Takahashi D., Matsushita J., Hozak P., Misteli T., Miyamoto K., Harata M. (2020). Impairment of nuclear F-actin formation and its relevance to cellular phenotypes in Hutchinson-Gilford progeria syndrome. Nucleus.

[41] Venit T., Semesta K., Farrukh S., Endara-Coll M., Havalda R., Hozak P., Percipalle P. (2020). Nuclear myosin 1 activates p21 gene transcription in response to DNA damage through a chromatin-based mechanism. Commun. Biol..

[42] Frottin F., Schueder F., Tiwary S., Gupta R., Körner R., Schlichthaerle T., Cox J., Jungmann R., Hartl F.U., Hipp M.S. (2019). The nucleolus functions as a phase-separated protein quality control compartment. Science.

[43] Azkanaz M., López A.R., de Boer B., Huiting W., Angrand P., Vellenga E., Kampinga H.H., Bergink S., Martens J.H.A., Schuringa J. (2019). Protein quality control in the nucleolus safeguards recovery of epigenetic regulators after heat shock. Elife.

[44] Zaffagnini G., Savova A., Danieli A., Romanov J., Tremel S., Ebner M., Peterbauer T., Sztacho M., Trapannone R., Tarafder A.K. (2018). Phasing out the bad-How SQSTM1/p62 sequesters ubiquitinated proteins for degradation by autophagy. Autophagy.

[45] Turco E., Witt M., Abert C., Bock-Bierbaum T., Su M.Y., Trapannone R., Sztacho M., Danieli A., Shi X., Fracchiolla D. (2019). FIP200 claw domain binding to p62 promotes autophagosome formation at ubiquitin condensates. Mol. Cell..

[46] Cohen-Kaplan V., Livneh I., Avni N., Cohen-Rosenzweig C., Ciechanove A. (2016). The ubiquitin-proteasome system and autophagy: Coordinated and independent activities. Int. J. Biochem. Cell. Biol..

[47] Latonen L., Moore H.M., Bai B., Jäämaa S., Laiho M. (2011). Proteasome inhibitors induce nucleolar aggregation of proteasome target proteins and polyadenylated RNA by altering ubiquitin availability. Oncogene.

[48] Kraft L.J., Manral P., Dowler J., Kenworthy A.K. (2016). Nuclear LC3 associates with slowly diffusing complexes that survey the nucleolus. Traffic.

[49] Salmina K., Huna A., Inashkina I., Belyayev A., Krigerts J., Pastova L., Vazquez-Martin A., Erenpreisa J. (2017). Nucleolar aggresomes mediate release of pericentric heterochromatin and nuclear destruction of genotoxically treated cancer cells. Nucleus.

[50] Mostofa M.G., Rahman M.A., Koike N., Yeasmin A.M., Islam N., Waliullah T.M., Hosoyamada S. (2018). CLIP and cohibin separate rDNA from nucleolar proteins destined for degradation by nucleophagy. J. Cell Biol..

[51] Iadevaia V., Zhang Z., Jan E., Proud C.G. (2012). mTOR signaling regulates the processing of pre-rRNA in human cells. Nucleic Acids Res..

[52] Tsang K.C., Liu H., Zheng X.F. (2010). mTOR binds to the promoters of RNA polymerase I- and III-transcribed genes. Cell. Cycle.

[53] Katagiri N., Kuroda T., Kishimoto H., Hayashi Y., Kumazawa T., Kimura K. (2015). The nucleolar protein nucleophosmin is essential for autophagy induced by inhibiting Pol I transcription. Sci. Rep..

[54] Marx B., Hufbauer M., Zigrino P., Majewski S., Markiefka B., Sachsenheimer T., Brügger B., Akgül B. (2018). Phospholipidation of nuclear proteins by the human papillomavirus E6 oncoprotein: Implication in carcinogenesis. Oncotarget.

