# Supplementary material for: Limited Proteolysis-Coupled Mass Spectrometry Identifies Phosphatidylinositol 4,5-Bisphosphate Effectors in Human Nuclear Proteome
Source: Cells. 2021 Jan 4;10(1):68. doi: 10.3390/cells10010068 (PMC7824793; doi:10.3390/cells10010068)
Supplement: Supplementary file 1 [file cells-10-00068-s001.zip › cells-1024842 Supplementary materials/Supplemetary materials.docx]

Supplementary materials

**Table S1.** Summary of all protein groups identified in this study. Protein groups identified by PIPsLiP-qMS are shown together with the results of statistical analysis and gene ontology annotation.

**Table S2.** Selected results from the gene ontology overrepresentation analysis. Selected GOBP and GOCC categories are shown with results of statistical evaluation.

**Table S3.** The PIP2-binding domain-containing proteins identified by bioinformatic analysis. The proteins containing canonical PIP2-binding domains identified by bioinformatic search are show with their annotation. .

**Table S4.** The summary of bioinformatic analysis of PIP2-binding motifs in ’exposed’ protein group. The results of bioinformatic search in ’exposed’ protein group indicating the position, type and occurrence frequency of KR motif.

**Table S5.** The summary of bioinformatic analysis of PIP2-binding motifs in ’hidden’ protein group. The results of bioinformatic search in ’hidden’ protein group indicating the position, type and occurrence frequency of KR motif.

**Table S6.** Summary of the most significant hits from the ‘exposed’ and ‘hidden’ proteins including the information about protein nuclear localization. The proteins from ’exposed’ and ’hidden’ groups identified by PIPsLiP-qMS with the manual evaluation of the nuclear localization based on uniprot.org and proteinatlas.org databases.

**Table S7.** The list of proteins annotated in GOBP as mRNA splicing, mRNA processing factors, and protein linked to nuclear speckles. Listed proteins are selected based on the GO analysis performed by Perseus software. The statistical significance is indicated.


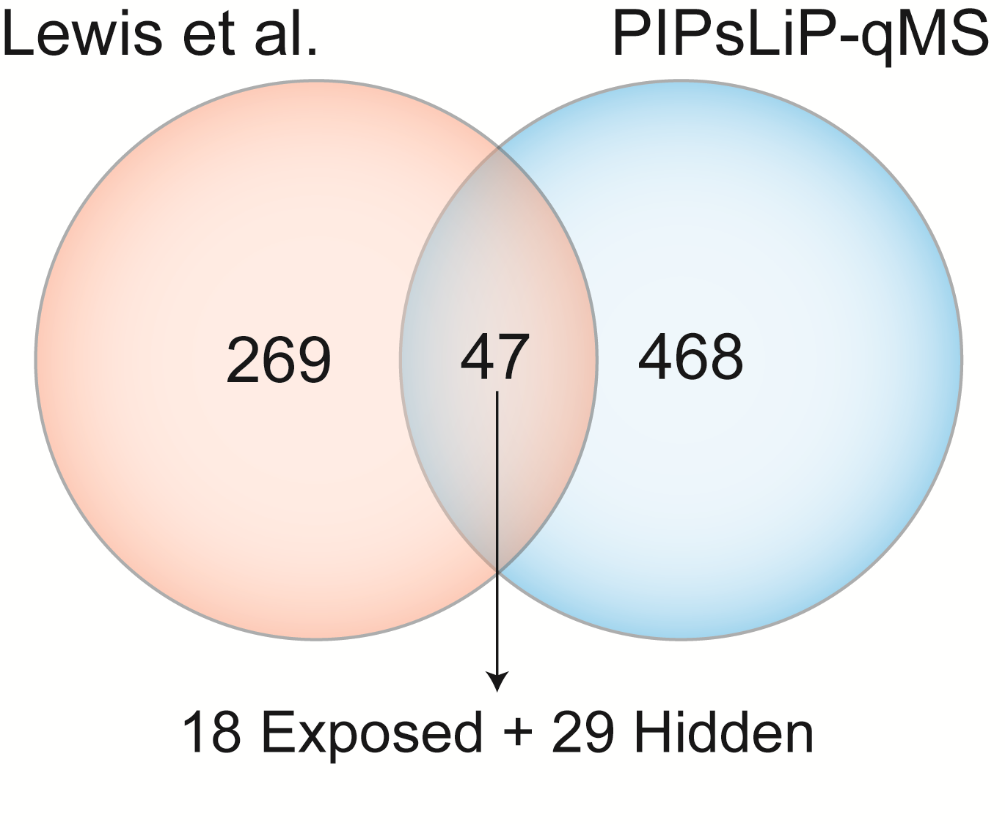


**Figure S1.** The Venn diagram representing the overlap of data provided by PIPsLiP-qMS with data presented in Lewis et al., 2011. Protein groups of ’exposed’ and “’hidden’” were cross-correlated with data from Lewis et al., 2011. The number of overlapping proteins which belongs to different groups is indicated.


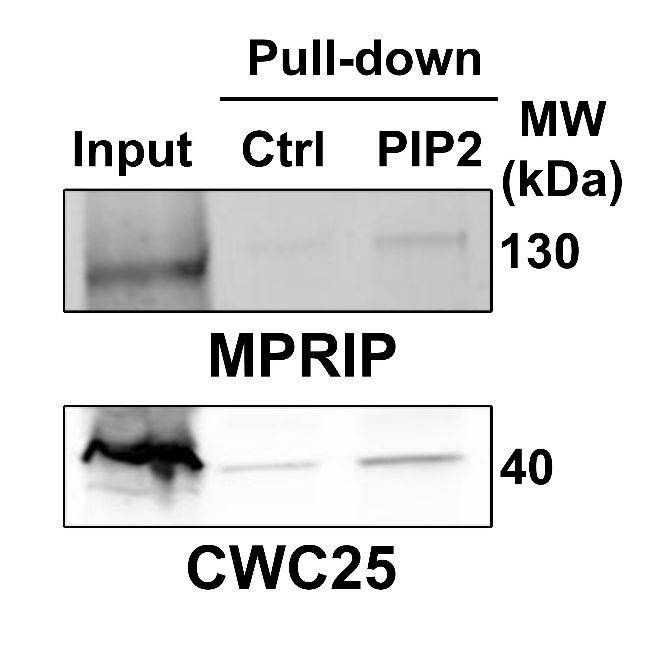


**Figure S2.** The verification of PIP2-binding capacity of MPRIP and CWC25. Input 2% of total protein added into pull-down binding assay. Ctrl - Control beads; PIP2-coated beads pull-down. For experimental detail please refer to section 2.13. Protein were detected by specific antibodies on western blot membrane (section *2.1.*).


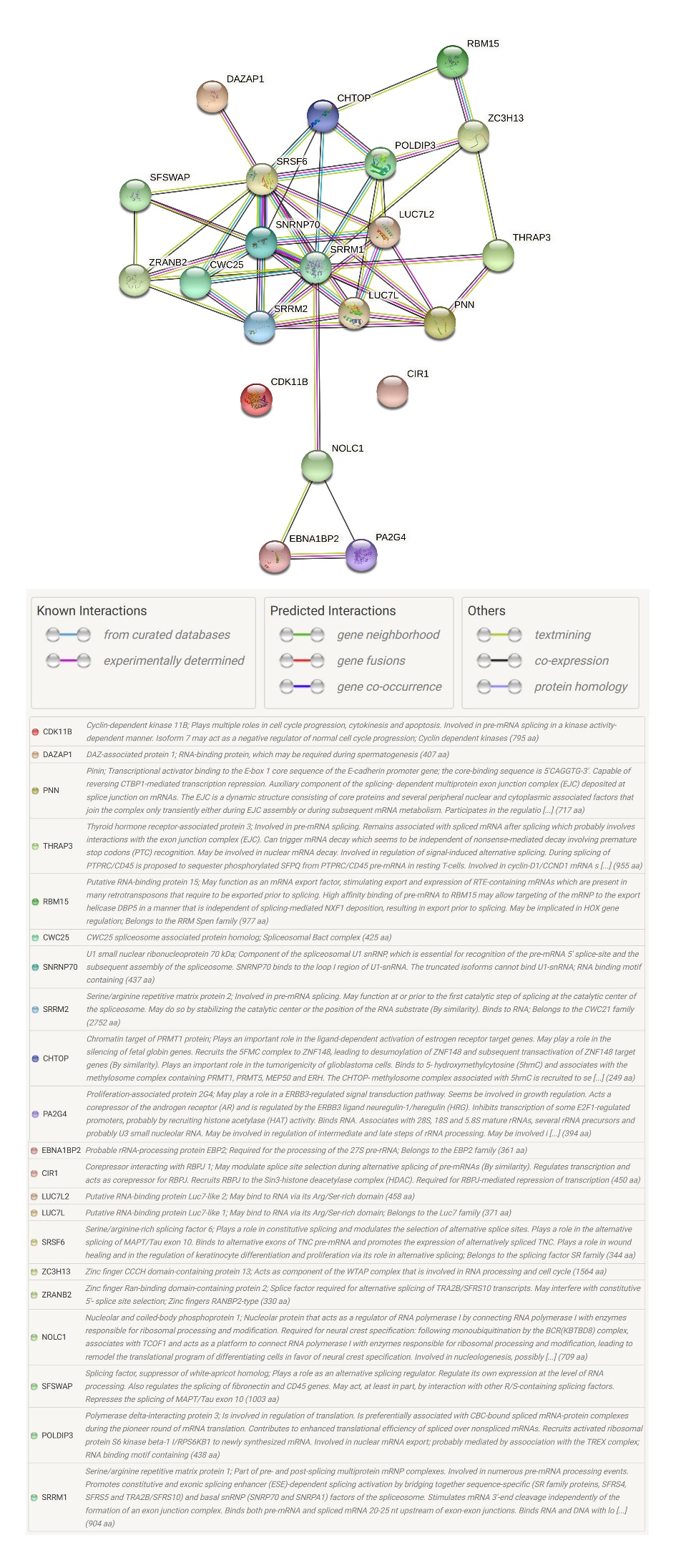


**Figure S3.** The Gene ontology analysis of ‘Exposed’ protein group using STRING.
