# Supplementary material for: Limited Proteolysis-Coupled Mass Spectrometry Identifies Phosphatidylinositol 4,5-Bisphosphate Effectors in Human Nuclear Proteome
Source: Cells. 2021 Jan 4;10(1):68. doi: 10.3390/cells10010068 (PMC7824793; doi:10.3390/cells10010068)
Supplement: Supplementary file 1 [file cells-10-00068-s001.zip › cells-1024842 Supplementary materials/Supplemetary materials.pdf]

cells

MDPI

Supplementary materials

Table S1. Summary of all protein groups identified in this study. Protein groups identified by PIPsLiP-qMS are shown together with the results of statistical analysis and gene ontology annotation.

Table S2. Selected results from the gene ontology overrepresentation analysis. Selected GOBP and GOCC categories are shown with results of statistical evaluation.

Table S3. The PIP2-binding domain-containing proteins identified by bioinformatic analysis. The proteins containing canonical PIP2-binding domains identified by bioinformatic search are show with their annotation. .

Table S4. The summary of bioinformatic analysis of PIP2-binding motifs in 'exposed' protein group. The results of bioinformatic search in 'exposed' protein group indicating the position, type and occurrence frequency of KR motif.

Table S5. The summary of bioinformatic analysis of PIP2-binding motifs in 'hidden' protein group. The results of bioinformatic search in 'hidden' protein group indicating the position, type and occurrence frequency of KR motif.

Table S6. Summary of the most significant hits from the 'exposed' and 'hidden' proteins including the information about protein nuclear localization. The proteins from 'exposed' and 'hidden' groups identified by PIPsLiP-qMS with the manual evaluation of the nuclear localization based on uniprot.org and proteinatlas.org databases.

Table S7. The list of proteins annotated in GOBP as mRNA splicing, mRNA processing factors, and protein linked to nuclear speckles. Listed proteins are selected based on the GO analysis performed by Perseus software. The statistical significance is indicated.

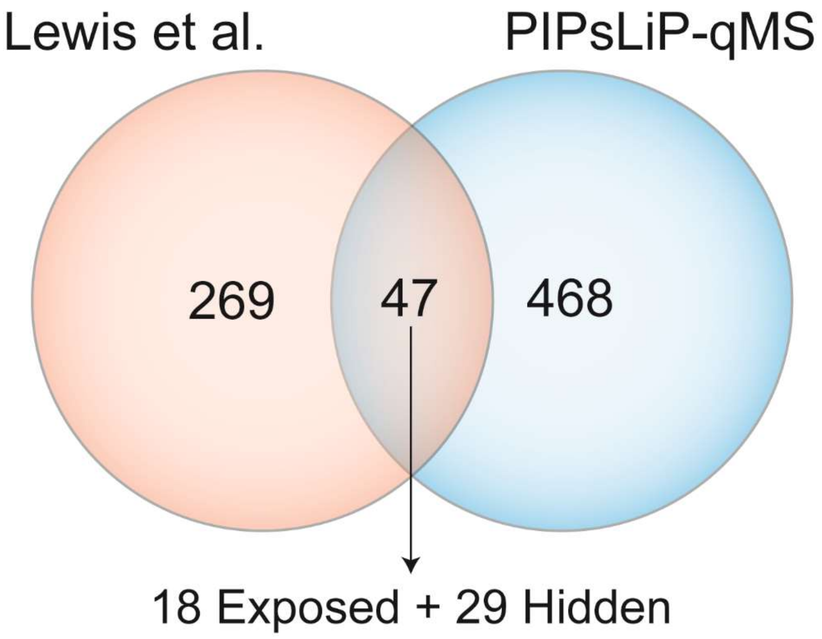

Figure S1. The Venn diagram representing the overlap of data provided by PIPsLiP-qMS with data presented in Lewis et al., 2011. Protein groups of 'exposed' and "'hidden'" were cross-correlated with data from Lewis et al., 2011. The number of overlapping proteins which belongs to different groups is indicated.

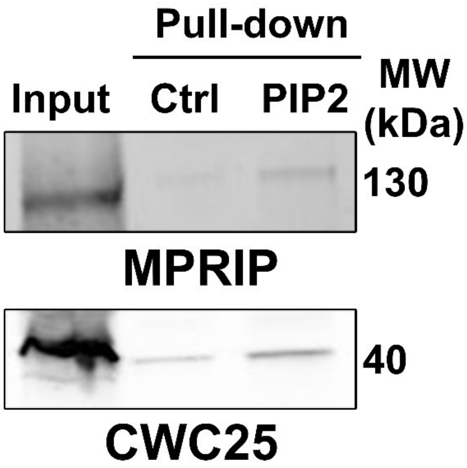

Figure S2. The verification of PIP2-binding capacity of MPRIP and CWC25. Input 2% of total protein added into pull-down binding assay. Ctrl - Control beads; PIP2-coated beads pull-down. For experimental detail please refer to section 2.13. Protein were detected by specific antibodies on western blot membrane (section *2.1.*).

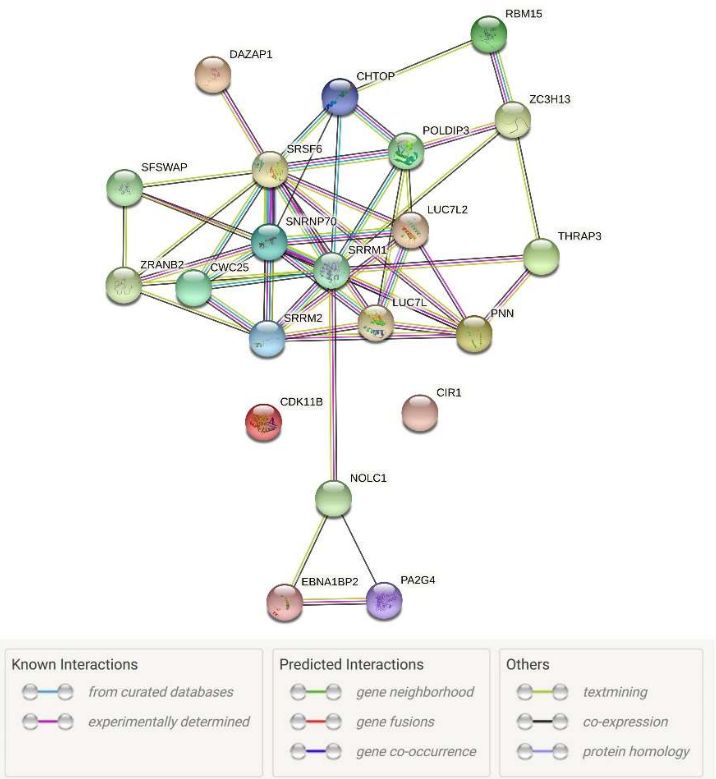

CDK11B	*Cyclin-dependent kinase 11B; Plays multiple roles in cell cycle progression, cytokinesis and apoptosis. Involved in pre-mRNA splicing in a kinase activity-dependent manner. Isofom 7 may act as a negative regulator of normal cell cycle progression; Cyclin dependent kinases (795 aa)*
DAZAP1	*DAZ-associated protein 1; RNA-binding protein, which may be required during spermatogenesis (407 aa)*
PNN	*Pinin; Transcriptional activator binding to the E-box 1 core sequence of the E-cadherin promoter gene; the core-binding sequence is 5'CAGGTG-3'. Capable of reversing CTBP1-mediated transcription repression. Auxiliary component of the splicing- dependent multiprotein exon junction complex (EJC) deposited at splice junction on mRNAs. The EJC is a dynamic structure consisting of core proteins and several peripheral nuclear and cytoplasmic associated factors that join the complex only transiently either during EJC assembly or during subsequent mRNA metabolism. Participates in the regulatio [...] (717 aa)*
THRAP3	*Thyroid hormone receptor-associated protein 3; Involved in pre-mRNA splicing. Remains associated with spliced mRNA after splicing which probably involves interactions with the exon junction complex (EJC). Can trigger mRNA decay which seems to be independent of nonsense-mediated decay involving premature stop codons (PTC) recognition. May be involved in nuclear mRNA decay. Involved in regulation of signal-induced alternative splicing. During splicing of PTPRC/CD45 is proposed to sequester phosphorylated SFPQ from PTPRC/CD45 pre-mRNA in resting T-cells. Involved in cyclin-D1/CCND1 mRNA s [...] (955 aa)*
RBM15	*Putative RNA-binding protein 15; May function as an mRNA export factor, stimulating export and expression of RTE-containing mRNAs which are present in many retrotransposons that require to be exported prior to splicing. High affinity binding of pre-mRNA to RBM15 may allow targeting of the mRNP to the export helicase DBP5 in a manner that is independent of splicing-mediated NXF1 deposition, resulting in export prior to splicing. May be implicated in HOX gene regulation; Belongs to the RRM Spen family (977 aa)*
CWC25	*CWC25 spliceosome associated protein homolog; Spliceosomal Bact complex (425 aa)*
SNRNP70	*U1 small nuclear ribonucleoprotein 70 kDa; Component of the spliceosomal U1 snRNP, which is essential for recognition of the pre-mRNA 5' splice-site and the subsequent assembly of the spliceosome. SNRNP70 binds to the loop I region of U1-snRNA. The truncated isoforms cannot bind U1-snRNA; RNA binding motif containing (437 aa)*
SRRM2	*Serine/arginine repetitive matrix protein 2; Involved in pre-mRNA splicing. May function at or prior to the first catalytic step of splicing at the catalytic center of the spliceosome. May do so by stabilizing the catalytic center or the position of the RNA substrate (By similarity). Binds to RNA; Belongs to the CWC21 family (2752 aa)*
CHTOP	*Chromatin target of PRMT1 protein; Plays an important role in the ligand-dependent activation of estrogen receptor target genes. May play a role in the silencing of fetal globin genes. Recruits the 5FMC complex to ZNF148, leading to desumoylation of ZNF148 and subsequent transactivation of ZNF148 target genes (By similarity). Plays an important role in the tumorigenicity of glioblastoma cells. Binds to 5- hydroxymethylcytosine (5hmC) and associates with the methylosome complex containing PRMT1, PRMT5, MEP50 and ERH. The CHTOP- methylosome complex associated with 5hmC is recruited to se [...] (249 aa)*
PA2G4	*Proliferation-associated protein 2G4; May play a role in a ERBB3-regulated signal transduction pathway. Seems be involved in growth regulation. Acts a corepressor of the androgen receptor (AR) and is regulated by the ERBB3 ligand neuregulin-1/heregulin (HRG). Inhibits transcription of some E2F1-regulated promoters, probably by recruiting histone acetylase (HAT) activity. Binds RNA. Associates with 28S, 18S and 5.8S mature rRNAs, several rRNA precursors and probably U3 small nucleolar RNA. May be involved in regulation of intermediate and late steps of rRNA processing. May be involved i [...] (394 aa)*
EBNA1BP2	*Probable rRNA-processing protein EBP2; Required for the processing of the 27S pre-rRNA; Belongs to the EBP2 family (361 aa)*
CIR1	*Corepressor interacting with RBPJ 1; May modulate splice site selection during alternative splicing of pre-mRNAs (By similarity). Regulates transcription and acts as corepressor for RBPJ. Recruits RBPJ to the Sin3-histone deacetylase complex (HDAC). Required for RBPJ-mediated repression of transcription (450 aa)*
LUC7L2	*Putative RNA-binding protein Luc7-like 2; May bind to RNA via its Arg/Ser-rich domain (458 aa)*
LUC7L	*Putative RNA-binding protein Luc7-like 1; May bind to RNA via its Arg/Ser-rich domain; Belongs to the Luc7 family (371 aa)*
SRSF6	*Serine/arginine-rich splicing factor 6; Plays a role in constitutive splicing and modulates the selection of alternative splice sites. Plays a role in the alternative splicing of MAPT/Tau exon 10. Binds to alternative exons of TNC pre-mRNA and promotes the expression of alternatively spliced TNC. Plays a role in wound healing and in the regulation of keratinocyte differentiation and proliferation via its role in alternative splicing; Belongs to the splicing factor SR family (344 aa)*
ZC3H13	*Zinc finger CCCH domain-containing protein 13; Acts as component of the WTAP complex that is involved in RNA processing and cell cycle (1564 aa)*
ZRANB2	*Zinc finger Ran-binding domain-containing protein 2; Splice factor required for alternative splicing of TRA2B/SFRS10 transcripts. May interfere with constitutive 5'- splice site selection; Zinc fingers RANBP2-type (330 aa)*
NOLC1	*Nucleolar and coiled-body phosphoprotein 1; Nucleolar protein that acts as a regulator of RNA polymerase I by connecting RNA polymerase I with enzymes responsible for ribosomal processing and modification. Required for neural crest specification: following monoubiquitination by the BCR(KBTBD8) complex, associates with TCOF1 and acts as a platform to connect RNA polymerase I with enzymes responsible for ribosomal processing and modification, leading to remodel the translational program of differentiating cells in favor of neural crest specification. Involved in nucleologenesis, possibly [...] (709 aa)*
SFSWAP	*Splicing factor, suppressor of white-apricot homolog; Plays a role as an alternative splicing regulator. Regulate its own expression at the level of RNA processing. Also regulates the splicing of fibronectin and CD45 genes. May act, at least in part, by interaction with other R/S-containing splicing factors. Represses the splicing of MAPT/Tau exon 10 (1003 aa)*
POLDIP3	*Polymerase delta-interacting protein 3; Is involved in regulation of translation. Is preferentially associated with CBC-bound spliced mRNA-protein complexes during the pioneer round of mRNA translation. Contributes to enhanced translational efficiency of spliced over nonspliced mRNAs. Recruits activated ribosomal protein S6 kinase beta-1 I/RPS6KB1 to newly synthesized mRNA. Involved in nuclear mRNA export; probably mediated by assoociation with the TREX complex; RNA binding motif containing (438 aa)*
SRRM1	*Serine/arginine repetitive matrix protein 1; Part of pre- and post-splicing multiprotein mRNP complexes. Involved in numerous pre-mRNA processing events. Promotes constitutive and exonic splicing enhancer (ESE)-dependent splicing activation by bridging together sequence-specific (SR family proteins, SFRS4, SFRS5 and TRA2B/SFRS10) and basal snRNP (SNRP70 and SNRPA1) factors of the spliceosome. Stimulates mRNA 3'-end cleavage independently of the formation of an exon junction complex. Binds both pre-mRNA and spliced mRNA 20-25 nt upstream of exon-exon junctions. Binds RNA and DNA with lo [...] (904 aa)*

Figure S3. The Gene ontology analysis of 'Exposed' protein group using STRING.
